# Profiling mitochondria-polyribosome lncRNAs associated with pluripotency

**DOI:** 10.1038/s41597-023-02649-3

**Published:** 2023-11-02

**Authors:** Lei Zhou, Hui Li, Tingge Sun, Xue Wen, Chao Niu, Min Li, Wei Li, Miguel A. Esteban, Andrew R. Hoffman, Ji-Fan Hu, Jiuwei Cui

**Affiliations:** 1 Key Laboratory of Organ Regeneration and Transplantation of Ministry of Education, Cancer Center, First Hospital of Jilin University, Changchun, Jilin 130061 P.R. China; 2 Laboratory of Integrative Biology, Guangzhou Institutes of Biomedicine and Health, Chinese Academy of Sciences, Guangzhou, China; 3 Stanford University Medical School, VA Palo Alto Health Care System, Palo Alto, CA 94304 USA

**Keywords:** Reprogramming, Epigenetics

## Abstract

Pluripotent stem cells (PSCs) provide unlimited resources for regenerative medicine because of their potential for self-renewal and differentiation into many different cell types. The pluripotency of these PSCs is dynamically regulated at multiple cellular organelle levels. To delineate the factors that coordinate this inter-organelle crosstalk, we profiled those long non-coding RNAs (lncRNAs) that may participate in the regulation of multiple cellular organelles in PSCs. We have developed a unique strand-specific RNA-seq dataset of lncRNAs that may interact with mitochondria (mtlncRNAs) and polyribosomes (prlncRNAs). Among the lncRNAs differentially expressed between induced pluripotent stem cells (iPSCs), fibroblasts, and positive control H9 human embryonic stem cells, we identified 11 prlncRNAs related to stem cell reprogramming and exit from pluripotency. In conjunction with the total RNA-seq data, this dataset provides a valuable resource to examine the role of lncRNAs in pluripotency, particularly for studies investigating the inter-organelle crosstalk network involved in germ cell development and human reproduction.

## Background & Summary

Long non-coding RNAs (lncRNAs) are transcripts of at least 200 nucleotides in length that lack a clear putative protein-coding ORF^1^. Although the number of characterized lncRNAs has dramatically increased, the biological roles of the lncRNAs in embryonic development, particularly in pluripotent reprogramming, have not been fully characterized. It was initially thought that lncRNAs were only present in the nucleus, but it is now clear that a number of lncRNAs encoded by the nucleus are also transported and localized to the cytoplasm^2^. LncRNAs found in the nucleus are usually related to epigenetic regulation at the transcriptional level. However, the finding that some lncRNAs are associated with cytoplasmic polyribosomes (prlncRNAs) suggests the coding potential^3^ for these RNAs. It is also possible that these cytoplasmic lncRNAs subserve a post-transcriptional regulatory function^4^ by fine-tuning the speed of translation or otherwise modifying the activity of ribosomes. Moreover, the base-pairing capability of prlncRNAs indicates that they can also interact with and regulate specific mRNAs^5^.

In addition to the numerous nuclear genome-encoded lncRNAs, the mitochondrial genome generates at least eight lncRNAs, several dsRNAs, and numerous small RNAs that either translocate into the cytosol and/or nucleus or remain in the mitochondria to perform various biological functions^6^. Three mitochondrial lncRNAs (mtlncRNAs), lncND5, lncCyt b, and lncND6, were identified using deep sequencing data from human HeLa cell mitochondria^7^. In both ischemic and non-ischemic human failing hearts, changes in the abundance of mtlncRNAs were noted in the left ventricle^8^. Mitochondrial function critically depends on the import of many nuclear-encoded macromolecules. In all eukaryotes, selected nuclear genome-encoded non-coding RNAs are partially redirected from the nucleus to the mitochondria, where they regulate mitochondrial gene expression^9^. These nuclear and mitochondrial genome-encoded lncRNAs may engage in inter-compartment crosstalk, either “nucleus-to-mitochondria” or “mitochondria-to-nucleus,” to maintain cellular homeostasis^10,11^. Aberrant shuttling of lncRNAs in this inter-compartment crosstalk is associated with human diseases, including cancer. For example, the mitochondria-encoded lncRNA lncCytB was located in mitochondria in normal hepatic cells. In hepatoma HepG2 cells, however, this lncRNA is considerably enriched in the nucleus^12^. In contrast, the nuclear genome-encoded lncRNA *MALAT1* is aberrantly transported to the mitochondria, where it acts as an epigenetic regulator to control metabolic reprogramming in hepatoma cells^13^. Thus, some lncRNAs may act as vital epigenetic messengers to coordinate the inter-organelle crosstalk.

Mammalian embryonic stem cells (ESCs) originate from the ectoderm of developing embryos and can differentiate into three germ layers. Induced PSCs (iPSCs) are derived from the direct reconstitution of somatic cells into ESC-like pluripotent cells via the introduction of specific transcription factors. The use of ESC and iPSCs in clinical treatments for tissue repair has prompted in-depth research into their biological characteristics^14^. However, the molecular mechanisms underlying stem cell differentiation remain unknown, and research on lncRNAs may shed new light on this process^15,16^. Forty lncRNAs were identified in mouse ESC PSCs. After knocking out 30 of them, mESCs were induced to differentiate into distinct lineages^17^. Chakraborty *et al*. identified three lncRNAs that maintained the pluripotent stem cell characteristics in mESCs and dubbed them pluripotency-related non-coding transcripts 1–3 (Panct1–3). After knocking out Panct1, the expression of pluripotency markers was decreased while the expression of lineage markers was increased^18^. Loewer *et al*. discovered that iPSCs are abundant in the intergenic long-chain non-coding RNA (lincRNA) ST8SIA3, named lincRNA reprogramming regulator (linc-ROR), which promotes the formation of iPSC clones by inhibiting pro-apoptotic pathways^19^.

Our group previously published a combined pluripotency-associated lncRNA dataset that covers the data of RNA reverse transcription-associated trap sequencing (RAT-seq), chromatin RNA *in situ* reverse transcription sequencing (CRIST-seq), and RNA-seq^20–25^. The integration of these datasets allowed us to identify many differentially expressed lncRNAs that are not only associated with pluripotency but also function as chromatin factors to regulate pluripotency. These lncRNAs epigenetically coordinate the pluripotency-regulatory network and regulate stem cell fate through various epigenetic mechanisms, including coordinating intrachromosomal looping, recruiting methyltransferases and demethylases, and activating eRNA pathway of stemness genes^20–25^. Some lncRNAs, like nuclear *Peln1*, use a novel *trans* mechanism to regulate the exit from pluripotency^21^.

However, the role of the lncRNAs involved in the inter-organelle regulatory network, including nuclear-mitochondrial-polyribosomal crosstalk, has not been characterized. This data descriptor presents a unique strand-specific RNA-seq dataset of prlncRNA and mtlncRNA from human iPSCs, H9 ESCs, and fibroblasts. This dataset provides a valuable resource for studying these inter-organelle lncRNAs and should provide the means of examining mechanisms underlying the regulation of germ cell development and human reproduction. Most importantly, these mitochondrial and polyribosomal RNA-seq data and total RNA-seq data may help define those lncRNAs that determine stem cell fate by coordinating inter-organelle epigenetic regulatory networks.

## Methods

### Characterization of iPSCs, H9 cells, and fibroblasts

**Fig. 1:**
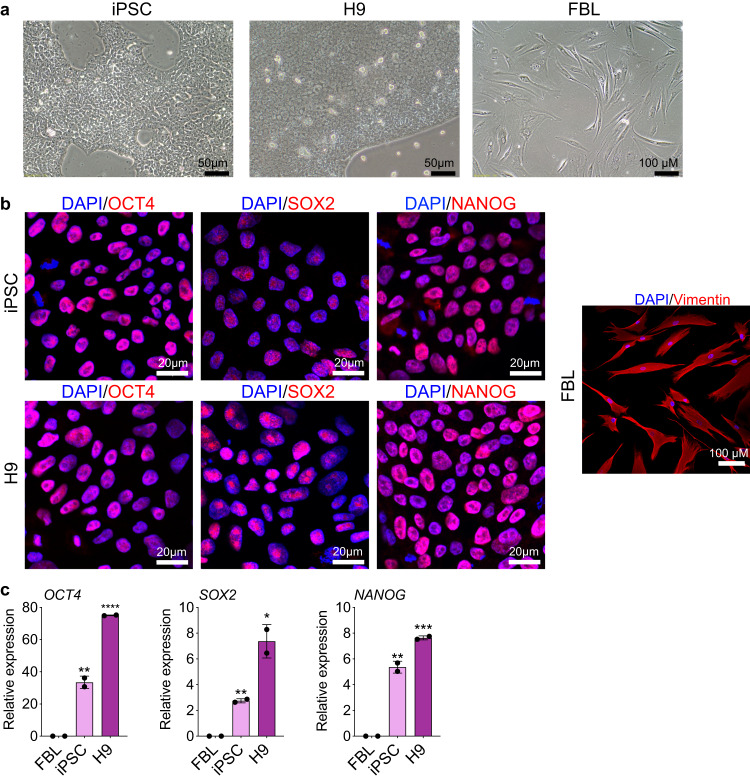
Characterization of iPSCs, H9 cells, and fibroblasts. (**a**) Representative morphology of cell lines used for RNA-seq. (**b**) Characteristics of cells by immunofluorescent staining with antibodies against biomarkers. The pluripotency of iPSCs and H9 ESCs was characterized by the positive staining of OCT4, SOX2, and NANOG stem cell markers. The terminally differentiated status of fibroblasts (FBL) was identified by the positive staining of vimentin. (**c**) Quantitative analysis of pluripotent biomarkers *OCT4*, *SOX2*, and *NANOG* by RT-qPCR.

Human embryonic stem cells (H9, WA09) were purchased from Wicell Research Institute (hPSCReg ID: WAe009-A). Skin fibroblasts (FBL, SPF7) were purchased from Coriell cell repository (AG06299) and cultured as described in previous studies^26,27^. Two iPSC cell lines (C11, S0730) were kindly provided by Professor Esteban of the Guangzhou Institutes of Biomedicine and Health, Chinese Academy of Sciences. They were induced from human urinary fibroblast using lentiviruses carrying the *OCT4, SOX2, KLF4*, and *c-MYC* as previously described^28^. The pluripotency of the cultured human iPSC and H9 PSCs were examined by morphology (Fig. 1a) and positive immunostaining of stem cell markers OCT4, SOX2, and NANOG (Fig. 1b). The terminally differentiated status of human fibroblasts was confirmed by positive staining of vimentin (Fig. 1b). Specifically, cells were fixed with 4% paraformaldehyde/PBS for 10–15 min, rinsed with PBS, then permeabilized and blocked with 0.1% Triton X-100/PBS containing 3% BSA for 30 min. After washing with PBS, cells were incubated first with antibodies against OCT4 (Abcam, ab19857), SOX2 (Abcam, ab97959), and NANOG (Invitrogen, PA1-097) at 4 °C overnight, followed by Alexa Flour 555 labelled secondary antibody (Invitrogen, A-21429) staining. After washing three times with PBS, samples were counterstained with 4′, 6-diamidino-2-phenylindole (DAPI, Invitrogen, D1306). Negative controls were stained without the use of primary antibodies. Fluorescence images were acquired with an Olympus FLUOVIEW FV3000. The pluripotency difference between the stem cells and fibroblasts was also confirmed by qPCR assay of stemness genes *OCT4*, *SOX2*, and *NANOG* (Fig. 1c).

### Sucrose gradient separation of polyribosomes and mitochondria

**Fig. 2:**
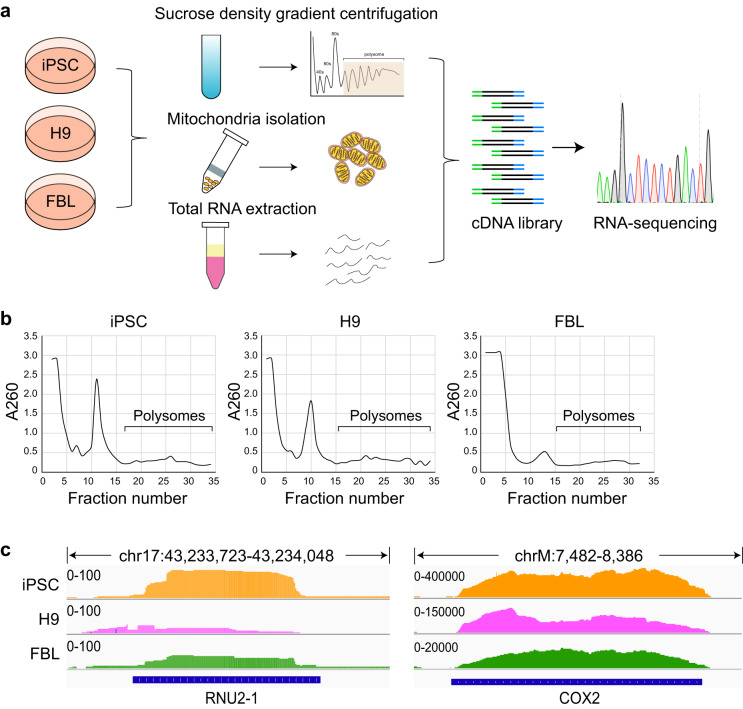
Schematic diagram of sample collection and mitochondrial RNA purity. (**a**) The schematic diagram of sample collection and RNA sequencing. The polysomes were separated by sucrose density gradient centrifugation, the mitochondria were isolated using Qproteome Mitochondria Isolation Kit (Qiagen, USA), and total RNAs were extracted with Trizol reagent. The RNAs were then reverse-transcribed for library sequencing. (**b**) Polysome fractions were collected from each sample. A260 absorbance profiles were used to determine the polysome fractions in the sucrose density gradients. (**c**) Mitochondrial RNA purity was determined by the enrichment of nuclear-located RNA *U2* and mitochondrial-located RNA *COX2*. The read counts in the RNA *U2* and *COX2* gene loci are shown by the bam files generated from the sequencing data.

To isolate polyribosomes, cell lysates were prepared after 10 min of cycloheximide treatment at 37 °C to stabilize translating polysomes, and sucrose gradient separation and fractionation were performed as previously described (Fig. 2a)^29^. The polysome fractions determined by 260 nm absorbance were pooled for expression analysis (Fig. 2b).

### Preparation of mitochondria

Mitochondria were prepared and purified using Qproteome Mitochondria Isolation Kit (Qiagen, USA) according to the manufacturer’s instructions. As previously reported^30^, 5 × 10^8^ cells were suspended in lysis buffer, incubated in ice for 10 min, and centrifuged at 1000 × g for 10 min at 4 °C. The pellet was rewashed with lysis buffer and resuspended in disruption buffer, followed by passing 10 times through a 24-gauge needle to ensure complete cell disruption and centrifuged at 1000 × g. The supernatant was centrifuged at 8000 rpm for 10 min at 4 °C to pellet the mitochondria. The mitochondria were washed and purified by adding them on top of layers of purification and disruption buffers. The solution was centrifuged at 13000 rpm for 15 min at 4 °C. The mitochondrial ring at the interface of purification buffer/disruption buffer was collected and washed in mitochondria storage buffer.

The purity of the mitochondrial RNAs (mtRNAs) was reflected by the expression ratio of mitochondrial RNA *COX2* and nuclear RNA *U2*. As shown in Fig. 2c, the read counts of *COX2* in the three types of cells were significantly higher than those of the U2 RNA, and the expression ratio of *COX2* and *U2* was ~200–5000.

### RNA extraction, cDNA library establishment, and Illumina sequencing

After pluripotency confirmation, Illumina RNA library sequencing was used to identify RNAs and lncRNAs that are differentially expressed in the reprogrammed cells (Fig. 2a). Total RNA was extracted using Trizol reagent (15596-018, Invitrogen, CA) according to the manufacturer’s instructions. The isolated RNAs were checked for RNA integrity by an Agilent Bioanalyzer 2100 (Agilent Technologies, CA, US). Total RNA was further purified by RNAClean XP Kit (A63987, Beckman Coulter, CA). RNase-Free DNase I (79254, QIAGEN, CA) was used to remove any contaminating DNA.

Ribosomal RNA was removed by a Ribo-Zero rRNA Removal Kit (#MRZH11124, Illumina, CA). RNAs were then fragmented into small pieces using a fragmentation reagent. The fragmented RNAs were subjected to first-strand cDNA synthesis using random hexamer-primed reverse transcription (18064014, SupperScript II reverse Transcriptase, Invitrogen, CA), followed by second-strand cDNA synthesis (Q32850, Qubit dsDNA HS Assay Kit, Invitrogen, CA). The cDNA fragments were 3′ adenylated and ligated with adaptors for PCR amplification for library construction. The library quality was assessed using Agilent2100. The libraries were clustered on an Illumina cBot Instrument and pair-sequenced using the Illumina NovaSeq 6000 platform.

### Raw read filtering and transcript mapping

The raw sequencing reads were subjected to fastp v0.20.0^31^ for removing: 1) adapter sequences in reads; 2) bases with a 3′ end Q value less than 20, indicating that the base error rate is greater than 0.01, where Q = −10logerror_ratio; 3) reads less than 25 in length; and 4) the ribosome RNA sequences of the species. The obtained clean reads were aligned to the human reference genome (GRCh38.p13) using the spliced mapping algorithm of StringTie^32^, which enables segmentation of reads that cannot be fully matched for mapping and is thus more suitable for eukaryotic transcriptome sequencing data containing intron regions. The alignments allowed for two mismatches; each read allowed for multiple hits < = 2, and the mapping generated BAM files.

The following software versions were used for quality control and data analysis: FastQC (v0.11.5): (https://www.bioinformatics.babraham.ac.uk/projects/fastqc/) QC filtering was performed using fastp 0.20.1 program with the default setting. (https://github.com/OpenGene/fastp). All reads were aligned to the human reference genome sequence by the STAR version 2.7.3a program. (https://github.com/alexdobin/STAR). Default parameters were used in the analyses.

### LncRNA identification

To identify known lncRNA, we used the Ensembl gene and transcript sequences, which have comprehensive annotation and detailed classification information of lncRNAs. In addition, we integrated other databases to verify the reliability of the lncRNAs. To evaluate the coding potential, lncRNAs were filtered using four different coding potential prediction algorithms including “Coding Potential Calculator 2”, “Coding-Non-Coding Identifying Tool”, “Coding-Potential Assessment Tool”, and “CPPred”. The novel lncRNAs were identified by taking the intersection of these four algorithms.

Cuffcompare in cufflinks (version: 2.2.1)^33^ was used to compare the mapping-derived annotations to the reference annotations to obtain novel lncRNA transcripts that did not match known annotated genes. Three types of transcripts (I, u, and x) were further extracted for lncRNA prediction, where i indicates transfrags falling entirely within a reference intron, u indicates unknown and intergenic transcripts, and x refers to exonic overlap with reference on the opposite strand. Then, transcripts with a length greater than or equal to 200 bp, more than two exons, and ORF less than 300 were chosen. Pfam^34^, the Coding Potential Calculator (CPC)^35^, and the Coding-Non-Coding Index (CNCI)^36^ were used for prediction, and the intersection of their predicted results was obtained. After removing known lncRNA sequences, transcripts that were not significant compared to Pfam and had CPC and CNCI scores less than 0 were designated as potential novel lncRNAs. The numbers of known and novel lncRNA and mRNA transcripts detected by each dataset are shown in Table 1.

**Table 1:** The numbers of known and novel lncRNA and mRNA transcripts detected by each dataset.

		lncRNA		mRNA
		Known	Novel	
iPSC	prRNA	3999	1457	31298
	mtRNA	10764	1942	53647
	Total RNA	13308	2222	57318
H9	prRNA	2306	572	23555
	mtRNA	10433	1862	55692
	Total RNA	11749	2080	55063
FBL	prRNA	9321	750	49703
	mtRNA	10350	1279	51486
	Total RNA	11497	1388	52461

### Expression abundance quantification

To standardize the expression level and make lncRNA expression levels between different samples comparable, we used cufflinks (version: 2.2.1)^33^ to convert the tophat mapping results to FPKM (Fragments Per Kilobase of Exon Model per Million Mapped Reads)^37^. The primary process is to obtain the precise location of the gene from the existing gene annotation file, then to count the reads covering the gene area, and finally to calculate the standardization of gene expression using the gene length and read count using the following formula:

$$FPKM=\frac{total\,exon\,Fragments}{mapped\,reads\,(Millions)\times exon\,length\,(KB)}$$

where total exon fragments refer to the number of fragments aligned to the gene exon (fragment: a pair of reads), exon length refers to the total length of the gene exon, and mapped reads refer to the total number of reads aligned to the reference genome.

### Differential expression analysis

Following quantification, the identification of differentially expressed lncRNAs (DE lncRNA) between different samples was performed using edgeR^38^, which can leverage the bootstraps of Kallisto to correct for technical variation. Multiple hypothesis testing was used to correct the obtained *p*-value, and the threshold was determined using False Discovery Rate^39,40^. The corrected *p*-value was then set as the *q*-value, and the statistical significance threshold was set to a *q*-value <  = 0.05 (−log10 *q*-value > 1.3). Simultaneously, we calculated the differential expression fold change in terms of the FPKM value and set the biological significance threshold to a minimum of a two-fold change. As a result, we defined DE lncRNAs as those with biological and statistical significance. The data of DE analysis of mtlncRNAs versus RNA-seq and prlncRNAs versus RNA-seq are shown in Figure S1. The lncRNAs that are significantly enriched in mtlncRNAs and prlncRNAs compared with total RNAs have been deposited in GEO dataset GSE216689^41^.

### Target gene prediction of DE lncRNAs

Since lncRNAs can regulate target gene expression at both the transcriptional and post-transcriptional levels, lncRNA target genes can be identified by analyzing the positional relationship (co-location) and expression correlation (co-expression) between lncRNAs and protein-coding genes^42^. The co-location method, for example, is based on the potential regulatory effect of lncRNA on nearby protein-coding genes. Therefore, target gene identification can be accomplished by searching for sequences within 100 kb upstream and downstream of lncRNA^43^. The co-expression analysis is based on the fact that certain lncRNAs can act on distant target genes. As a result, identifying its target genes is accomplished by correlating the expression of different gene products. Generally, this analysis is performed when the sample size exceeds five^44^. Due to the small sample size in this study, only the co-location prediction results are presented.

### GO and KEGG enrichment analysis

Gene Ontology (GO) enrichment analyses of target genes of differentially expressed lncRNAs were implemented by the GOseq^45,46^. The specific principle is to map the selected DE lncRNA-targeted genes to each term of the GO database to calculate the number of genes contained in each entry. The hypergeometric test was then used to identify significantly enriched GO terms (with a corrected *p*-value < 0.05) enriched by DE lncRNAs-targeted genes. KEGG (http://www.genome.jp/kegg/) is a database resource for understanding high-level functions and utilities of a biological system, such as the cell, the organism, and the ecosystem, from molecular-level information, especially large-scale molecular datasets generated by genome sequencing and other high throughput experimental technologies. We used KOBAS software^47^ to test the statistical enrichment of DE lncRNAs-targeted genes in KEGG pathways. The GO and KEGG enrichment of differentially-expressed RNA transcripts was deposited in NCBI GEO databases (GSE216689)^41^.

### Data records

The sequencing data in the fastQ format have been deposited in NCBI GEO databases (GSE216689)^41^ The FastQ format data will serve as the raw sequencing data for further downstream processing. The processed data (bedgraph), the general transfer format (gtf) file, the FPKM values and the genome locations of all detected transcripts have been deposited in NCBI Gene Expression Omnibus (GSE216689)^41^.

## Technical Validation

### Quality control of RNA samples and library

The quality of RNA samples prepared from H9, iPSC, and fibroblasts was determined using the Agilent Bioanalyzer 2100 (Agilent Technologies). Each sample had an RNA integrity number greater than 7.0, indicating that the values met the requirements for an RNA-sequencing library. The library quality was checked using Agilent2100, producing an average of 370–380 bp fragments, including adapters.

To further validate the quality of these datasets, we compared the abundance of lncRNAs found in polyribosomes that have been reported in a colon cancer cell line^4^ and in a hepatocellular carcinoma cell line^48^. As seen from the FPKM data in Figure S2a,b, lncRNAs *CASC7* and *TUG1* were abundantly enriched in polyribosomal RNA-seq datasets as compared with the total RNA-seq dataset. Similarly, *COX2* and *ND5* were abundant in mitochondrial RNA-seq (Figs. S3a,b). In addition, we also used RT-qPCR to validate the abundance of these lncRNAs in isolated polyribosomal RNA and mitochondrial RNA samples (Figs. S4, S5).

### Quality control of sequencing data and DE lncRNAs

**Fig. 3:**
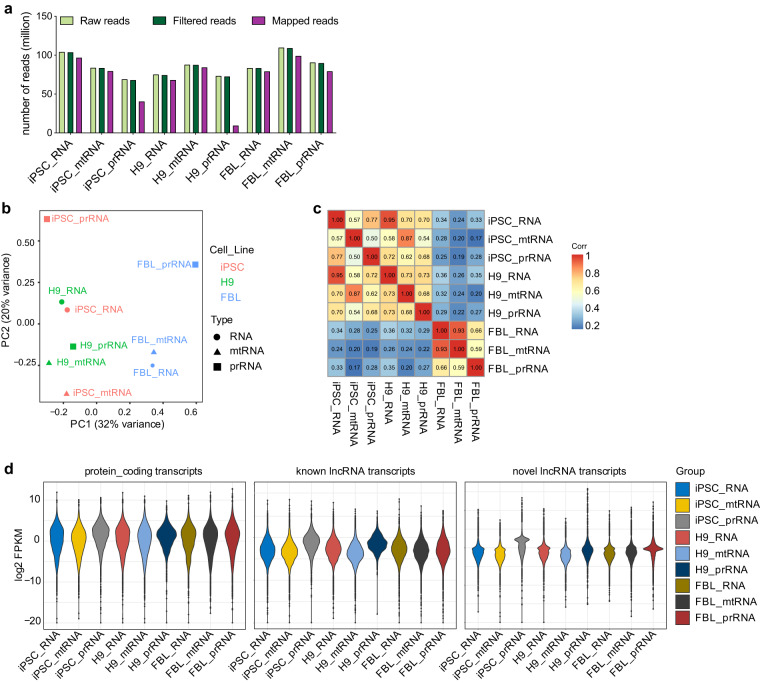
Differential expression analysis of RNA sequencing. (**a**) Quality control of RNA-sequencing data. (**b**) Principal component analysis (PCA) of transcripts in RNA-sequencing. (**c**) Heat map of transcript level correlation distance between samples. (**d**) Expression levels of protein-coding, known lncRNA, and novel lncRNA transcripts.

We applied FastQC v0.11.5 software to determine sequencing data quality. The per base sequence quality was high, with a median quality score above 30. The pattern of GC composition was similar to the theoretical distribution, indicating that the samples were free from contamination. In addition, the sequence length distribution also corresponded to the theoretical curve. The sequencing on Illumina NovaSeq 6000 generated mitochondria-associated RNA raw reads and polyribosome-associated RNA raw reads for H9, iPSC, and fibroblasts, respectively. After removing low-quality reads, clean reads were obtained for H9 (87,491,656), iPSC (83,502,776), and fibroblast (109,026,364) mitochondria-associated RNAs, respectively. At the same time, polyribosome-associated RNA clean reads were also obtained for H9 (72,565,674), iPSC (67,917,382), and fibroblasts (89,788,274). After Seqtk filtering, a total of 84,225,062 (96.27%), 79,386,203 (95.07%) and 99,030,086 (90.83%) clean reads were generated for H9, iPSC, and fibroblast mitochondria-associated RNA, as well as 9,479,552 (13.06%), 40,462,642 (59.58%), and 79,180,606 (88.19%) clean reads for H9, iPSC, and fibroblasts polyribosome-associated RNA (Fig. 3a). These reads were then mapped to the human genome (GRCh38.p13) for lncRNAs using the STAR software^49^.

To evaluate between-group differences and within-group sample duplication, we conducted principal component analysis (PCA) (Fig. 3b). PCA is a mathematical dimensionality reduction process that uses an orthogonal transformation to convert a set of linearly related variables into a set of linearly uncorrelated new variables, also known as principal components, to display the data in a lower dimension feature. It is possible to maintain as much information as possible in the variables and limit the number of variables as little as possible by using PCA, simplifying both the calculation and the interpretation of the findings. Additionally, the PCA analysis can be utilized to identify the main component with the greatest contribution as the data representative for the results visualization.

Based on quantitative and differential expression analyses, Pearson’s correlation coefficients of the transcript expression level of each sample showed that H9 and C11 iPS cells had high similarity in transcript expression, while FBL cells had significant differences from the two kinds of pluripotent cells (Fig. 3c). The expression levels of different kinds of transcripts, including protein-coding, known lncRNA, and novel lncRNA, are shown in Fig. 3d.

### Identification of novel pluripotency-associated polysomal lncRNAs

By integrating the polyribosomal RNA-seq and total RNA-seq data, we identified 11 novel lncRNAs from the top differentially expressed transcripts that were upregulated in both pluripotent stem cells H9 and C11. These RNA transcripts did not have known gene IDs and gene names, and they had higher FPKM in H9 and iPSC prRNAs than that in FBL. They were thus named PARIT (pluripotency-associated ribosome-interacting transcripts) 1–11 (Table 2). These lncRNAs were among the most upregulated prRNA transcripts between iPSC and FBL, as well as between H9 and FBL.

**Table 2:** Information of identified polyribosome-associated lncRNAs.

Name	Transcript ID	Transcript locus	Strand	Length (nt)
*PARIT1*	MSTRG.7050.1	ch12:14707462–14710251	Forward	1124
*PARIT2*	MSTRG.17555.1	ch2:7872749–7873255	Reverse	507
*PARIT3*	MSTRG.9727.1	ch14:41515929–41521760	Forward	271
*PARIT4*	MSTRG.19654.1	ch2:192920671–192920874	Forward	204
*PARIT5*	MSTRG.32410.1	ch8:79389062–79389304	Reverse	243
*PARIT6*	MSTRG.28377.1	ch6:35563168–35566992	Reverse	726
*PARIT7*	MSTRG.11840.1	ch15:88560871–88561087	Reverse	217
*PARIT8*	MSTRG.25127.1	ch4:91657384–91657750	Reverse	367
*PARIT9*	MSTRG.20551.1	ch20:29299450–29299703	Reverse	254
*PARIT10*	MSTRG.26528.1	ch5:71850915–71851115	Forward	201
*PARIT*11	MSTRG.25787.4	ch4:179166528–179170251	Reverse	3599

**Fig. 4:**
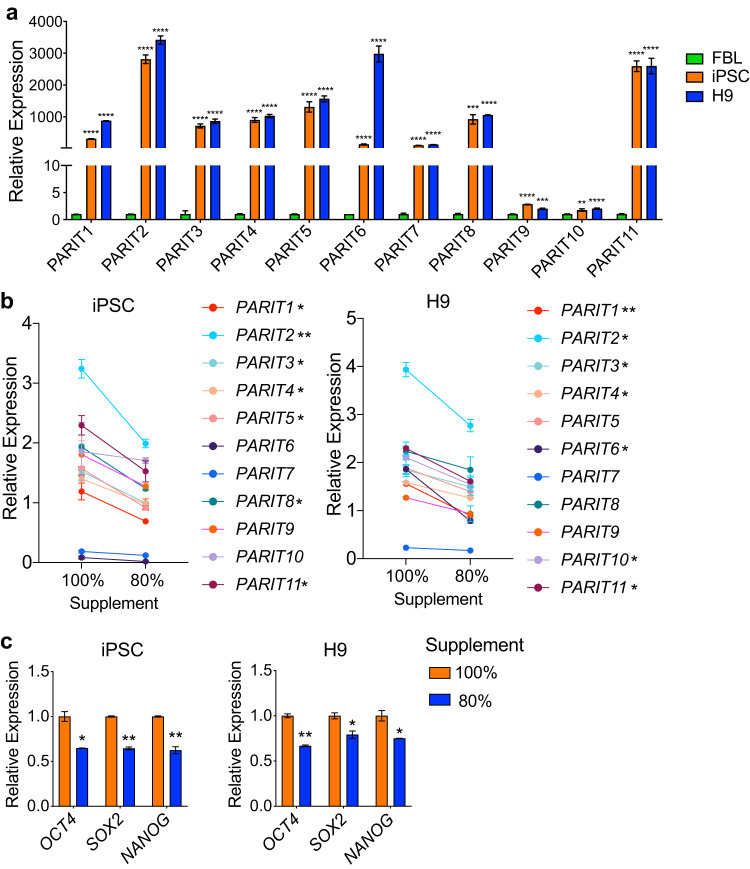
Identified novel lncRNAs that are associated with pluripotency. (**a**) RT-qPCR quantitation of lncRNAs PARIT1-11 in the polyribosome fraction between iPSCs and H9 cells and fibroblasts. Data are standardized over internal control β-Actin and are calculated as fold-changes of iPSCs and H9 cells over FBL. The experiment was performed in duplicate. (**b**) Downregulation of PARIT1-11 in cell differentiation. iPSCs and H9 cells were cultured with mTeSR1 supplemented (100%) according to the manufacturer’s instructions to maintain stemness or cultured with 80% supplement and 20% FBS to induce cell differentiation. The experiment was performed in duplicate. *p < 0.05 and **p < 0.01 compared with the 100% supplement group. (**c**) Downregulation of stemness genes in cell differentiation. The stemness genes *OCT4*, *SOX2* and *NANOG* were detected by RT-qPCR during cell differentiation induced by culturing with 80% supplement and 20% FBS. The experiment was performed in duplicate. *p < 0.05 and **p < 0.01 compared with 100% supplement group.

We then used RT-qPCR to confirm differential expression of these lncRNAs in the polyribosome fraction between H9/C11 and FBL cells (Fig. 4a) using specific primers (Table 3). The correlation between PARIT1-11 expression and stem cell pluripotency was validated by a cell differentiation test in C11 cells, introduced by replacing 20% of the supplement in the mTESR1 medium with FBS. The expression of PARIT1-11 was reduced with the addition of FBS, as were the stemness genes of *OCT4*, *SOX2* and *NANOG*, as shown in Fig. 4b,c. Currently, we know very little about the function of these prlncRNAs and mtlncRNAs. Future studies are needed to explore the role of these lncRNAs using organelle-specific targeting approaches.

**Table 3:** Primers for lncRNAs.

Name	Sequence	Size (nt)
PARIT1-F	CAAGGAGGGAGTAGAGGTATCT	137
PARIT1-R	CTGCTTCCCTGACTATTCCTGGA	
PARIT2-F	CCAGATGGCCTGAAGTAACT	113
PARIT2-R	TTAAGGCAAGGACCGGCCAT	
PARIT3-F	GCCTGAAGTAACTGAAGAATC	125
PARIT3-R	CACAAGGTAATGTCATCAGT	
PARIT4-F	ACTGTTGTGGGTATTGATGGC	116
PARIT4-R	ACTGGGCAGGTGGGGATAACT	
PARIT5-F	GAGGCTACCCACTCCACGTTA	128
PARIT5-R	TGTCTAAGTTGGCACCAGAGT	
PARIT6-F	ACAGTTACTGTGGTGAGCAG	130
PARIT6-R	TCCCAAGCTGGTTCAAGCTT	
PARIT7-F	TGACCTCTTATGTCTGCACC	120
PARIT7-R	CATAGTGAAGGAAGCAAGCCT	
PARIT8-F	GTATTGACGGCCAGGCTTCTA	123
PARIT8-R	CTCAGCCTAATAAGGGAACTG	
PARIT9-F	ATTACCCACTCCCTACTCGG	139
PARIT9-R	GCAGCTGCTGGCACCAGACTT	
PARIT10-F	TGCGGTAACGTGACCGATCC	161
PARIT10-R	CAGCGAGAGCTCACCGGATG	
PARIT11-F	AGTGGTCCCAATTCTTAGACC	100
PARIT11-R	ATTGGTGATGGCCTGGATACG	
OCT4-F	TCGAGAACCGAGTGAGAGG	124
OCT4-R	GAACCACACTCGGACCACA	
SOX2-F	ATGACCAGCTCGCAGACCTAC	106
SOX2-R	TTGACCACCGAACCCATGGAG	
NANOG-F	AGGCAAACAACCCACTTCT	278
NANOG-R	TCACACCATTGCTATTCTTCG	
CASC7-F	AGGTACCACCTGGTGGATAAGG	123
CASC7-R	GCGCAGAGTGTCTTGGTGAACC	
TUG1-F	CTCACAAGGCTGCACCAGATT	146
TUG1-R	TTACTCTGGGCTTCTGCACAGT	
COX2-F	ATGGCACATGCAGCGCAAGTAGGTC	134
COX2-R	GTTAGGAAAAGGGCATACAGGAC	
ND5-F	GCTGCTAGGAGGAGGCCTAGTAG	140
ND5-R	TACAACCGTATCGGCGATATCG	
GAPDH-F	GTCAAGGCTGAGAACGGGAA	158
GAPDH-R	AAATGAGCCCCAGCCTTCTC	
U2-F	ATCTGTTCTTATCAGTTTAATATCTG	151
U2-R	GGGTGCACCGTTCCTGGAGGTAC	

### Supplementary information

Supplementary Information

## Data Availability

No custom code was generated for this work.
