# Supplementary material for: Profiling mitochondria-polyribosome lncRNAs associated with pluripotency
Source: Sci Data. 2023 Nov 2;10:755. doi: 10.1038/s41597-023-02649-3 (PMC10622415; doi:10.1038/s41597-023-02649-3)
Supplement: Supplementary file 1 — Supplementary Information [file 41597_2023_2649_MOESM1_ESM.pdf]

Supplementary contents:

Figure S1. LncRNA DE analysis of mtRNAs vs. total RNA and prRNAs vs. total RNA

Figure S2. The FPKM of polyribosome-enriched lncRNA CASC7 and TUG1 in three prRNA-seq datasets

Figure S3. The FPKM of mitochondrial RNA COX2 and ND5 in three mtRNA-seq datasets

Figure S4. Validation of polysome-associated lncRNAs by RT-qPCR

Figure S5. Validation of mitochondrial RNAs by RT-qPCR

Supplemental Figure legends

Figure S1. LncRNA DE analysis of mtRNAs vs. total RNA and prRNAs vs. total RNA sequencing datasets.

a. Volcano plots of lncRNA transcripts in iPSC mtRNA-seq versus total RNA-seq and prRNA-seq versus total RNA-seq.
b. Volcano plots of lncRNA transcripts in H9 mtRNA-seq versus total RNA-seq and prRNA-seq versus total RNA-seq.
c. Volcano plots of lncRNA transcripts in FBL mtRNA-seq versus total RNA-seq and prRNA-seq versus total RNA-seq.

Figure S2. The FPKM of polyribosome-enriched lncRNA *CASC7* and *TUG1* in three prRNA-seq datasets.

a. The *CASC7* FPKM. The FPKM values of *CASC7* in fibroblast (FBL), iPSC (C11) and H9 polyribosomal RNA sequencing (prRNA-seq) and total RNA sequencing (RNA-seq) showed that lncRNA *CASC7* was enriched in polyribosomal RNAs.
b. The *TUG1* FPKM. The FPKM values of *TUG1* in FBL, iPSC and H9 prRNA-seq and total RNA-seq showed that lncRNA *TUG1* was enriched in polyribosomal RNAs.

Figure S3. The FPKM of mitochondrial RNA *COX2* and *ND5* in three mtRNA-seq datasets.

a. The *COX2* FPKM. The FPKM values of *COX2* in fibroblast (FBL), iPSC (C11) and H9 mitochondrial RNA sequencing (mtRNA-seq) showed that *COX2* mRNA was enriched in mitochondrial RNAs.

b. The *ND5* FPKM. The FPKM values of *ND5* in FBL, iPSC and H9 mtRNA-seq showed that *ND5* mRNA was enriched in mitochondrial RNAs.

Figure S4. Validation of polyribosome-associated lncRNAs by RT-qPCR.

a. The enrichment of lncRNA *CASC7* in polyribosomal RNA (prRNA) and total RNA samples. Data were standardized over that of internal control *GAPDH*.
b. The enrichment of lncRNA *TUG1* in polyribosomal RNA (prRNA) and total RNA samples. Data were standardized over that of *GAPDH*.

The experiment was performed in duplicate. *$p < 0.05$, ** $p < 0.01$, *** $p < 0.005$, and **** $p < 0.001$ as compared with total RNAs. Consistent with the FPKM data, the qPCR quantitation also supports the accumulation of *CASC7* and *TUG1* in polyribosomal RNAs.

Figure S5. Validation of mitochondrial RNAs by RT-qPCR

a. The enrichment of *COX2* in mitochondrial RNA (mtRNA) and total RNA samples. Data were standardized over that of internal control *GAPDH*.
b. The enrichment of *ND5* in mitochondrial RNA (mtRNA) and total RNA samples. Data were standardized over that of *GAPDH*.

The experiment was performed in duplicate. *$p < 0.05$, ** $p < 0.01$, *** $p < 0.005$, and **** $p < 0.001$ as compared with total RNAs. Consistent with the FPKM data, these qPCR data also suggest the mitochondrial accumulation of *COX2* and *ND5*.

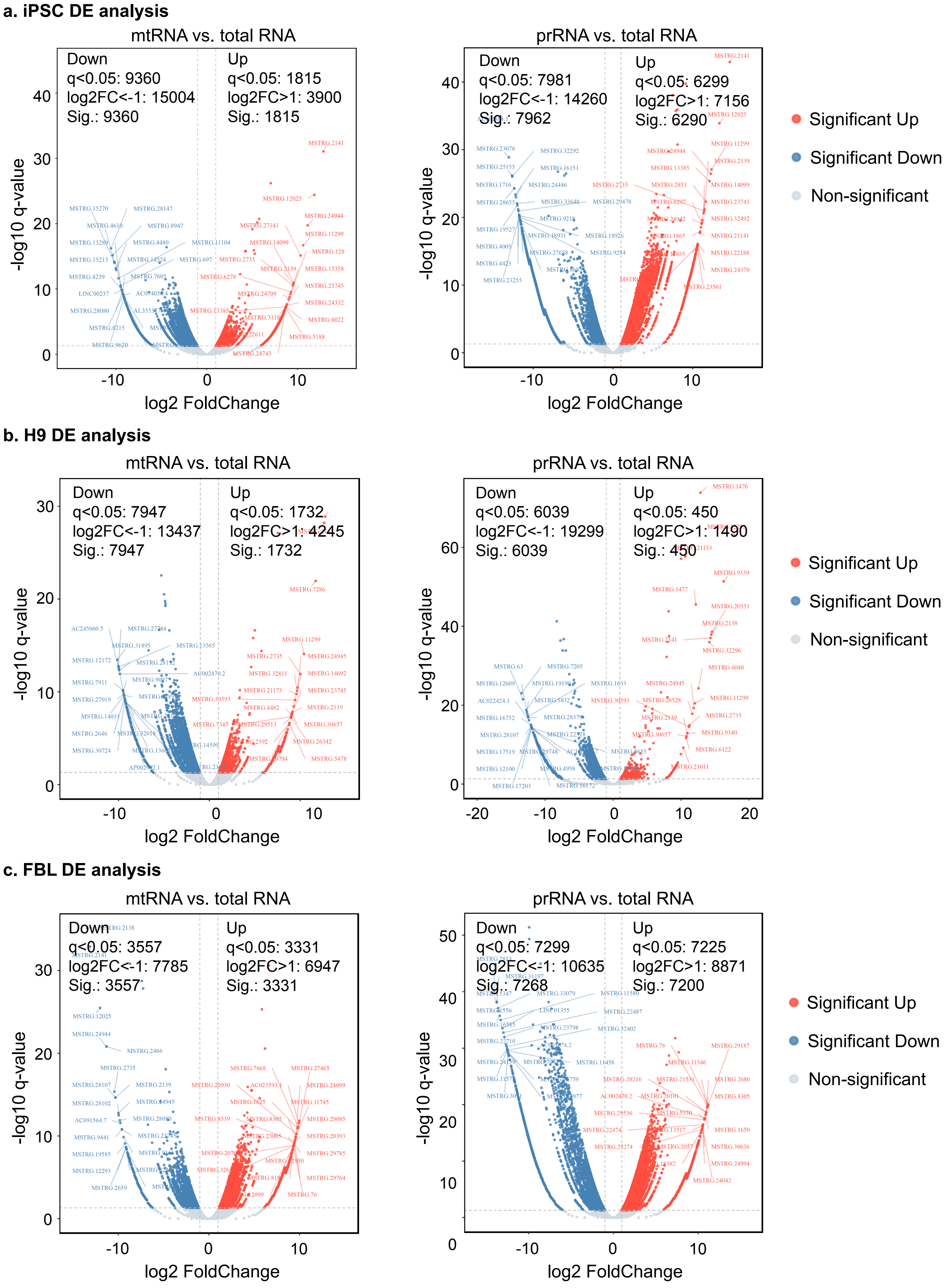

Figure S1. LncRNA DE analysis of mtRNAs vs. total RNA and prRNAs vs. total RNA

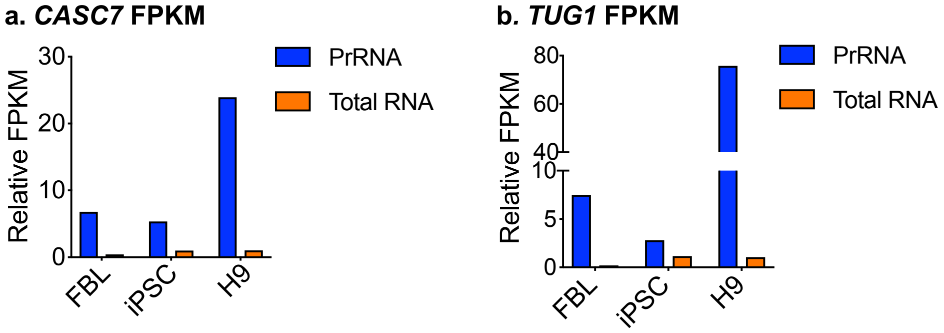

Figure S2. The FPKM of polyribosome-enriched lncRNA *CASC7* and *TUG1* in three prRNA-seq datasets

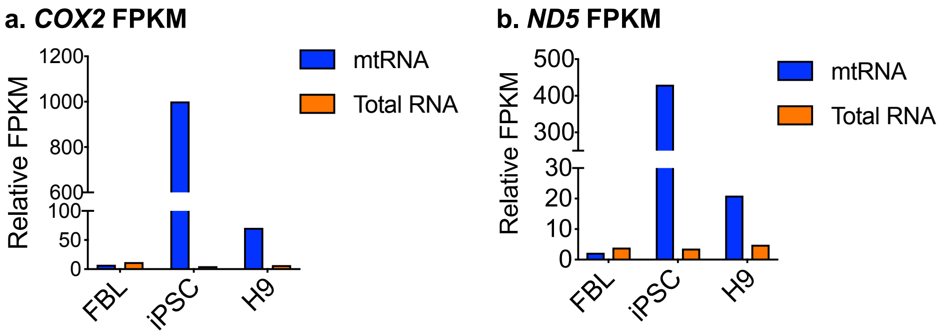

Figure S3. The FPKM of mitochondrial RNA *COX2* and *ND5* in three mtRNA-seq datasets

a. *CASC7* in polysomal RNAs

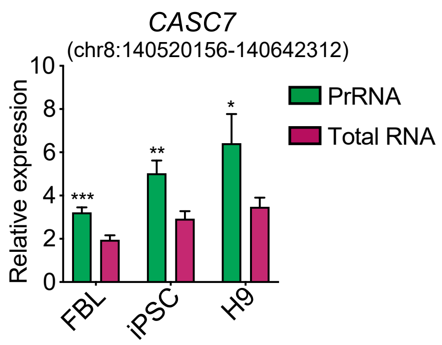

b. *TUG1* in polysomal RNAs

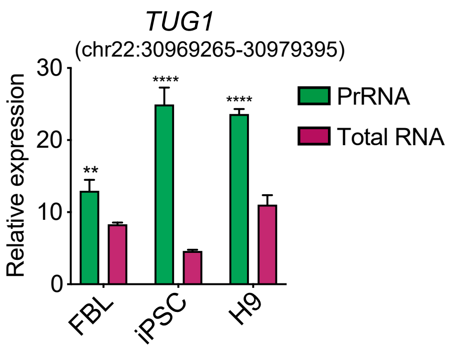

Figure S4. Validation of polysome-associated lncRNAs by RT-qPCR

a. *COX2* in mitochondrial RNAs **b. *ND5* in mitochondrial RNAs**

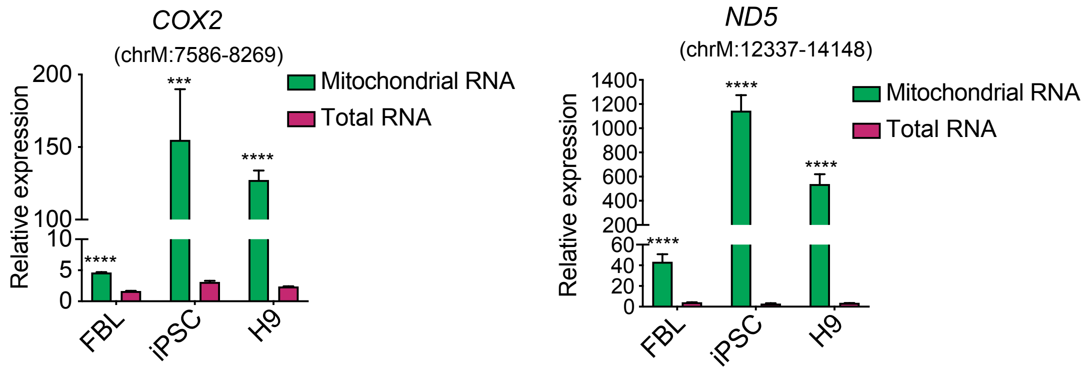

Figure S5. Validation of mitochondrial RNAs by RT-qPCR
